# The effects of L-carnitine supplementation on glycemic control: a systematic review and meta-analysis of randomized controlled trials

**DOI:** 10.17179/excli2019-1447

**Published:** 2019-08-19

**Authors:** Hadis Fathizadeh, Alireza Milajerdi, Željko Reiner, Fariba Kolahdooz, Zatollah Asemi

**Affiliations:** 1 Department of Microbiology, Kashan University of Medical Sciences, Kashan, Iran; 2 Students' Scientific Research Center, Tehran University of Medical Sciences, Tehran, Iran; Department of Community Nutrition, School of Nutritional Sciences and Dietetics, Tehran University of Medical Sciences, Tehran, Iran; 3 Department of Internal Medicine, University Hospital Centre Zagreb, School of Medicine, University of Zagreb, Zagreb, Croatia; 4 Indigenous and Global Health Research, Department of Medicine, University of Alberta, Edmonton, Canada; 5 Research Center for Biochemistry and Nutrition in Metabolic Diseases, Kashan University of Medical Sciences, Kashan, Iran

**Keywords:** L-carnitine, glycemic control, insulin resistance, meta-analysis

## Abstract

The findings of trials investigating the effect of L-carnitine administration on glycemic control are controversial. This meta-analysis of randomized controlled trials (RCTs) was performed to explore the effects of L-carnitine intake on glycemic control. Two authors independently searched electronic databases including MEDLINE, EMBASE, Cochrane Library, Web of Science, PubMed and Google scholar from 1990 until February 2019, in order to find relevant RCTs. 37 studies with 44 effect sizes met the inclusion criteria and were eligible for the meta-analysis. L-carnitine supplementation resulted in a significant reduction in fasting plasma glucose (FPG) (WMD: -4.57; 95 % CI: -6.88, -2.25), insulin (WMD: -1.21; 95 % CI: -1.85, -0.57), homeostatic model assessment for insulin resistance (HOMA-IR) (WMD: -0.67; 95 % CI: -0.90, -0.44) and HbA1C concentrations (WMD: -0.30; 95 % CI: -0.47, -0.13). L-Carnitine supplementation significantly reduced FPG, insulin, HOMA-IR, and HbA1c levels.

## Abbreviations

FPG: Fasting Plasma Glucose; HOMA-IR: Homeostatic Model Assessment for Insulin Resistance; HbA1c: Hemoglobin A1C

## Introduction

There are many definitions of metabolic syndrome (MetS) but most of them include three of five cardiovascular disease (CVD) risk factors: hypertriglyceridemia, low levels of high density lipoprotein-cholesterol (HDL-C), abdominal obesity, hypertension, and hyperglycemia (Murthy et al., 2016[37]). MetS is related with an increased risk of CVD, non-alcoholic fatty liver disease (NAFLD), type 2 diabetes mellitus (T2DM) and subsequent mortality (Ford, 2005[15]; Gami et al., 2007[18]; Tabák et al., 2009[48]). As a consequence of lack of physical activity and excessive energy intake, MetS has become a disease which affects more than 25 % of the world population and causes serious concerns worldwide (Grundy, 2016[21]). Insulin resistance and disturbed glucose metabolism resulting with hyperglycemia are most important in development of T2DM and other diseases related to MetS (Grundy et al., 2005[22]).

Carnitine is a dipeptide which is an essential factor for the membrane transport of acyl-coenzyme A (CoA) (Suzuki et al., 1982[47]). Carnitine deficiency reduces the use of lipids which causes serious metabolic defects (Takenaka et al., 2007[49]). Many studies have found that treatment with carnitine has a substantial role in glucose tolerance, weight loss, fatty acids metabolism and insulin function (Molfino et al., 2010[34]; Zhang et al., 2014[55]). A relatively recently published meta-analysis by Xu et al. (2017[52]), indicated that carnitine supplementation has beneficial effect in patients with insulin resistance. Several randomized controlled trials (RCTs) have investigated the efficacy of carnitine on markers related to glycemic control in patients with MetS, but their results are inconsistent. Alipour et al. (2014[3]) reported that 8 weeks of carnitine supplementation in obese diabetic women on hypocaloric diet significantly reduced fasting glucose and insulin resistance. A 8-week carnitine supplementation in patients on peritoneal dialysis improved insulin sensitivity (Bonomini et al., 2013[9]). It has also been shown that carnitine administration at a dosage of 2 g/day for 12 months to subjects with T2DM resulted in a significant improvement in homeostatic model assessment-insulin resistance (HOMA-IR) (Derosa et al., 2011[12]). However, Shirali et al. (2016[44]) indicated that L-carnitine plus caffeine in male teen soccer players increased their fasting glucose levels. Liang et al.(1998[27]) suggested that 3 g/day carnitine supplementation during 12 weeks to non-insulin dependent diabetes mellitus patients had no effects on fasting glucose, HbA1c, and insulin levels.

Discrepancies between the studies might be due to different concepts of studies as well as different formulations and dosages of carnitine used. The aim of this paper is to review systematically the trials investigating the effect of L-carnitine supplementation on glycemic control and to perform a meta-analysis in order to determine the effects of L-carnitine on markers related to glycemic control.

## Methods

### Search strategy

Two authors independently searched electronic databases including MEDLINE, EMBASE, Cochrane Library, Web of Science, PubMed and Google scholar databases from 1990 until February 2019 for relevant RCTs investigating the association between L-carnitine intake and glycemic control. The search strategy was limited to RCTs conducted in English databases and performed in humans. The following MeSH and keywords were used to identify primary RCTs: intervention ("L-carnitine" OR "propionyl L-Carnitine" OR "Acetyl-L-carnitine" OR "carnitine orotate complex" OR "L-carnitine -L-tartrate" AND "supplementation" OR "intake"), and parameters ["fasting plasma glucose (FPG)" OR "insulin" OR "homeostasis model assessment of insulin resistance (HOMA-IR)" OR "HbA1C"]. The reference lists of related RCTs and previous reviews were hand-reviewed to detect further studies which were not captured in primary search.

### Inclusion and exclusion criteria

RCTs fulfilling the following criteria were included in meta-analysis: trials on humans with cross-over design and/or either parallel, data analyzing the effect of L-carnitine on glycemic parameters extracted from RCTs with standard deviation (SD) and 95 % confidence interval (CI) for both treatment and control groups. Other studies such as *in vitro* studies, animal experiments, observational studies, studies with duration below two weeks, case reports and studies without a control group were excluded from this meta-analysis.

### Data extraction and quality assessment

Two authors independently (HF and AM) screened the articles based on the inclusion criteria. As the first step the title and abstract of studies were reviewed. Any disagreement was resolved by the judgment of the third author (ZA). 

The following data were extracted from selected studies: year of publication, the first authors' name, study location and design, dosage of intervention, sample size, duration of study, age of subjects, type of disease, the mean and SD for glycemic control in each treatment group. The quality of the selected RCTs was assessed by same authors independently using the Cochrane Collaboration risk of bias tool based on the following criteria: "allocation concealment, randomization generation, outcome assessors and blinding of participants, selective outcome reporting, incomplete outcome data, and other sources of bias". 

### Data synthesis and statistical analysis

Effects of L-carnitine on the changes of glycemic parameters. Weighted mean difference (WMD) with 95 % CI was used for pooling data to determine effect sizes. The random-effect model was used to report the pooled effect sizes using 95 % CI.

### Heterogeneity and publication bias

Heterogeneity of included studies was evaluated using Cochrane's Q test and I-square test. The funnel plot, as well as the Beggs's and Egger's regression tests was used to determine the publication bias. STATA 11.0 (Stata Corp., College Station, TX) was applied for data analysis.

## Results

### Characteristics of included studies 

**Figure 1:**
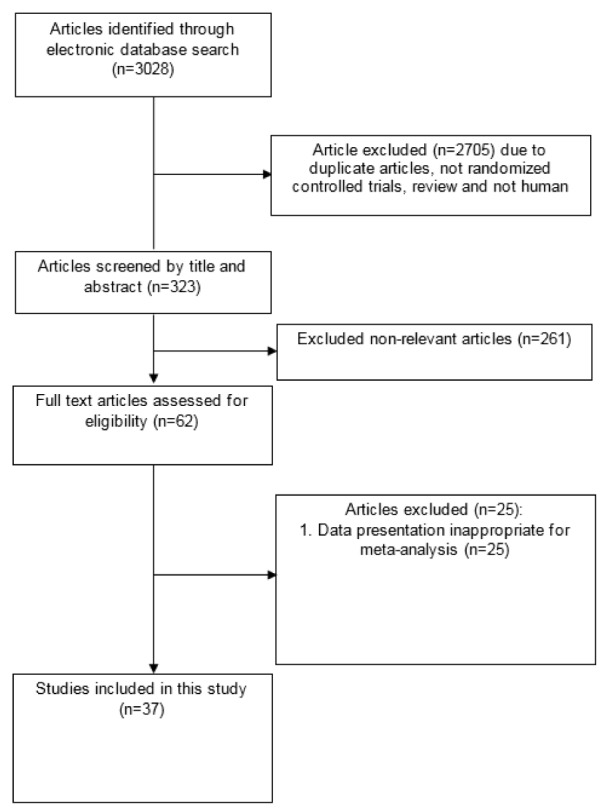
Literature search and review flowchart for selection of studies

Diagram for study selection is shown in Figure 1(Fig. 1). 37 studies with 44 effect sizes were included in this systematic review and meta-analysis (Table 1(Tab. 1); References in Table 1: Akbarzadeh et al., 2016[1]; Alavinejad et al., 2016[2]; Alipour et al., 2014[3]; An et al., 2016[4]; Bae et al., 2015[5]; Bañuls et al., 2015[6]; Bloomer et al., 2009[7]; Bloomer et al., 2009[8]; Bonomini et al., 2013[9]; Derosa et al., 2003[10]; Derosa et al., 2010[11]; Derosa et al., 2010[13]; El-sheikh et al., 2019[14]; Galvano et al., 2009[17]; Ghorbani et al., 2017[19]; Gonzalez-Ortiz et al., 2008[20]; Hlais et al., 2012[24]; Hong et al., 2014[25]; Liang et al., 1998[27]; Malaguarnera et al., 2007[28]; Malaguarnera et al., 2009[30]; Malaguarnera et al., 2009[31]; Malaguarnera et al., 2010[29]; McMackin et al., 2007[33]; Morano et al., 2007[35]; Mosah et al., 2015[36]; Parvanova et al., 2018[38]; Rafraf et al., 2012[39]; Rahbar et al., 2005[40]; Rondanelli et al., 2013[41]; Samimi et al., 2016[42]; Santo et al., 2006[43]; Shirali et al., 2016[44]; Soare et al., 2014[45]; Wall et al., 2011[50]; Yonei et al., 2007[54]; Yonei et al., 2008[53]). The studies were published between 1998 and 2018. A total of 2467 subjects, including 1243 persons in intervention and 1224 persons in control groups, were recruited in these studies. Studies were done in China, Iran, Italy, India, USA, Japan, Mexico, UK, Lebanon, Korea, Spain, Egypt and Iraq. Studies used L-carnitine, propionyl L-carnitine, glycine propionyl L-carnitine, and acetyl L-carnitine for treatment. The dosages varied between 200 to 3,000 mg/day, with a duration range between 23 days and 12 months.

### The effects of L-carnitine supplementation on FPG

**Figure 2:**
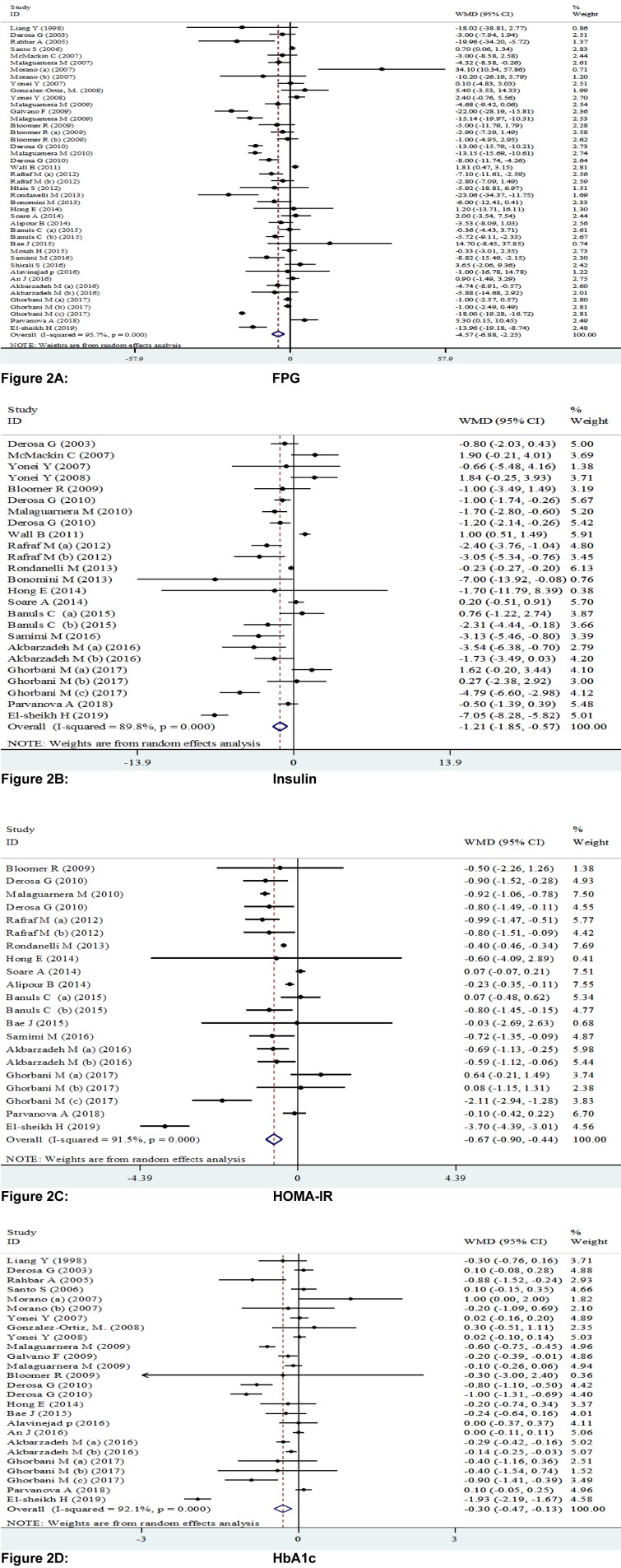
Figure 2A-D: Meta-analysis of glycemic control weighted mean difference estimates for A) FPG, B) insulin, C) HOMA-IR, D) HbA1c in the L-carnitine supplements and placebo groups (CI=95 %). Different capital letters indicate various dosage of L-carnitine used and different phases of L-carnitine treatment

Pooling 44 effect sizes from 37 studies, a significant reduction was found in FPG following L-carnitine supplementation (WMD): -4.57; 95 % CI: -6.88, -2.25) (Table 2(Tab. 2) and Figure 2A(Fig. 2); References in Figure 2: Akbarzadeh et al., 2016[1]; Alavinejad et al., 2016[2]; Alipour et al., 2014[3]; An et al., 2016[4]; Bae et al., 2015[5]; Bañuls et al., 2015[6]; Bloomer et al., 2009[7]; Bloomer et al., 2009[8]; Bonomini et al., 2013[9]; Derosa et al., 2003[10]; Derosa et al., 2010[11]; Derosa et al., 2010[13]; El-sheikh et al., 2019[14]; Galvano et al., 2009[17]; Ghorbani et al., 2017[19]; Gonzalez-Ortiz et al., 2008[20]; Hlais et al., 2012[24]; Hong et al., 2014[25]; Liang et al., 1998[27]; Malaguarnera et al., 2007[28]; Malaguarnera et al., 2009[30]; Malaguarnera et al., 2009[31]; Malaguarnera et al., 2010[29]; McMackin et al., 2007[33]; Morano et al., 2007[35]; Mosah et al., 2015[36]; Parvanova et al., 2018[38]; Rafraf et al., 2012[39]; Rahbar et al., 2005[40]; Rondanelli et al., 2013[41]; Samimi et al., 2016[42]; Santo et al., 2006[43]; Shirali et al., 2016[44]; Soare et al., 2014[45]; Wall et al., 2011[50]; Yonei et al., 2007[54]; Yonei et al., 2008[53]). This finding remained unchanged in most subgroup analyses. However, it became non-significant in studies done on participants aged <45 years (WMD: -0.55; 95 % CI: -1.43, 0.33), studies used L-carnitine in dosages of 1,000-2,000 mg/day (WMD: -0.24; 95 % CI: -1.53, 2.00), and those with a duration of 3-6 months (WMD: -0.20; 95 % CI: -0.76, 0.37), as well as studies done in healthy persons (WMD: -0.29; 95 %CI: -1.16, 0.59). A significant increase in FPG was found in two available studies on patients with renal diseases (WMD: 0.63; 95 % CI: 0.00, 1.27) (Table 3(Tab. 3)). 

### The effects of L-carnitine supplementation on insulin levels

Combining 25 effect sizes from 20 studies, we found a significant reductive effect of L-carnitine supplementation on insulin levels (WMD: -1.21; 95 % CI: -1.85, -0.57) (Table 2(Tab. 2) and Figure 2B(Fig. 2)). No significant changes in insulin concentrations were found in studies which enrolled <50 subjects (WMD: 0.01; 95 % CI: -0.37, 0.39), studies with a duration of 6-12 months (WMD: -0.28; 95 % CI: -0.61, 0.04), and those done on patients with CVD (WMD: -0.85; 95 % CI: -2.07, 0.37) (Table 3(Tab. 3)). 

### The effects of L-carnitine supplementation on HOMA-IR

The pooled analysis of data from 16 studies with 21 effect sizes showed a significant reduction in HOMA-IR following intake of L-carnitine supplements (WMD: -0.67; 95 % CI: -0.90, -0.44) (Table 2(Tab. 2) and Figure 2C(Fig. 2)). This finding remained unchanged in all subgroup analyses (Table 3(Tab. 3)).

### The effects of L-carnitine supplementation on HbA1C

L-carnitine supplementation resulted in a significant reduction in HbA1C concentrations, when we combined data from 22 studies with 26 effect sizes (WMD: -0.30; 95 % CI: -0.47, -0.13) (Table 2(Tab. 2) and Figure 2D(Fig. 2)). This finding did not change through subgroup analyses (Table 3(Tab. 3)).

## Discussion

This is the first meta-analysis of RCTs analyzing the effect of carnitine supplementation on glucose homeostasis parameters. In the present study, we showed that carnitine supplementation can significantly reduce FPG, insulin, HOMA-IR, and HbA1c levels.

Previous evidence suggested that carnitine might have a positive impact on glycemic control. A meta-analysis by Xu et al. (Xu et al., 2017[52]) indicated that carnitine supplementation had beneficial effects on HOMA-IR score. Supplementation with carnitine at a dosage of 2 g/day for 4 weeks in patients with impaired glucose metabolism was associated with a significant reduction of insulin levels and HOMA-IR score. In a trial by Bae et al. (2015[5]), carnitine supplementation resulted with a significant decrease in fasting glucose, HbA1c, and HOMA-IR score. Opposite findings were also reported. For example, Liang et al. (1998[27]) were unable to find any significant effects of carnitine on FPG, HbA1c, or insulin levels. 

Different dosages of carnitine, different types of carnitine as well as the use of only carnitine or carnitine combined with other supplements, heterogeneity in design of studies, and differences in populations involved in studies are some of the possible reasons that may explain different results. When patients with T2DM have other risk factors, particularly components of MetS, they are at especially high risk for CVD (Grundy et al., 2004[23]). In order to prevent CVD, control of blood glucose and other CVD risk factors in these patients is very important (Gæde et al., 2003[16]). Due to changed insulin secretion and pancreatic beta cell function normal glucose equilibrium is in this condition impaired (Xia et al., 2012[51]). Several mechanisms have been suggested as possible explanations for beneficial effects of carnitine on fasting glucose and insulin resistance. Increasing mitochondrial oxidation of long-chain acyl-CoAs is one of important mechanisms by which carnitine could improve glycemic control (Kerner and Hoppel, 2000[26]; McGarry and Brown, 1997[32]). It has been shown that carnitine supplementation reduces oxidative stress in diabetic patients (Malaguarnera et al., 2009[30]). Carnitine supplementation may have beneficial effects on glucose homeostasis also by modulating the expression of genes involved in insulin signaling pathway, changing the expression of gluconeogenic and glycolytic enzymes, regulating the intra-mitochondrial acyl-CoA/CoA ratio, and modulating the activity of pyruvate dehydrogenase complex (Steiber et al., 2004[46]). 

## Conclusions

This meta-analysis has shown that L-carnitine supplementation significantly reduces FPG, insulin, HOMA-IR, and HbA1c levels.

## Acknowledgements

Not applicable.

## Funding

Not applicable.

## Competing interests

The authors declare no conflict of interest.

## Figures and Tables

**Table 1:**
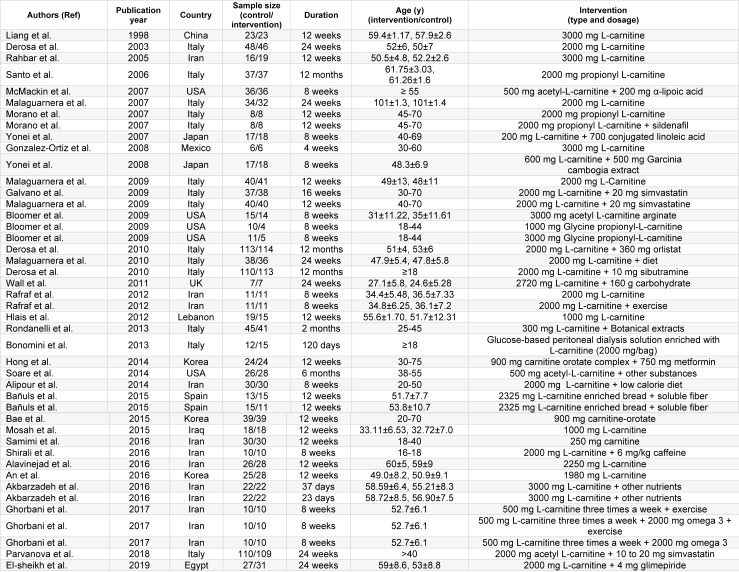
Characteristics of included primary clinical trials

**Table 2:**
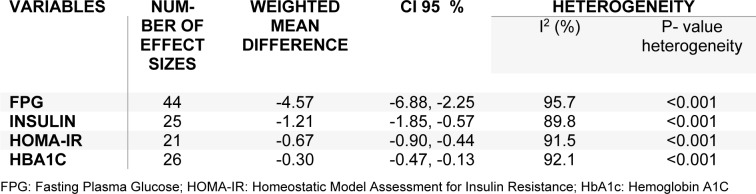
The effects of carnitine supplementation on glycemic control

**Table 3:**
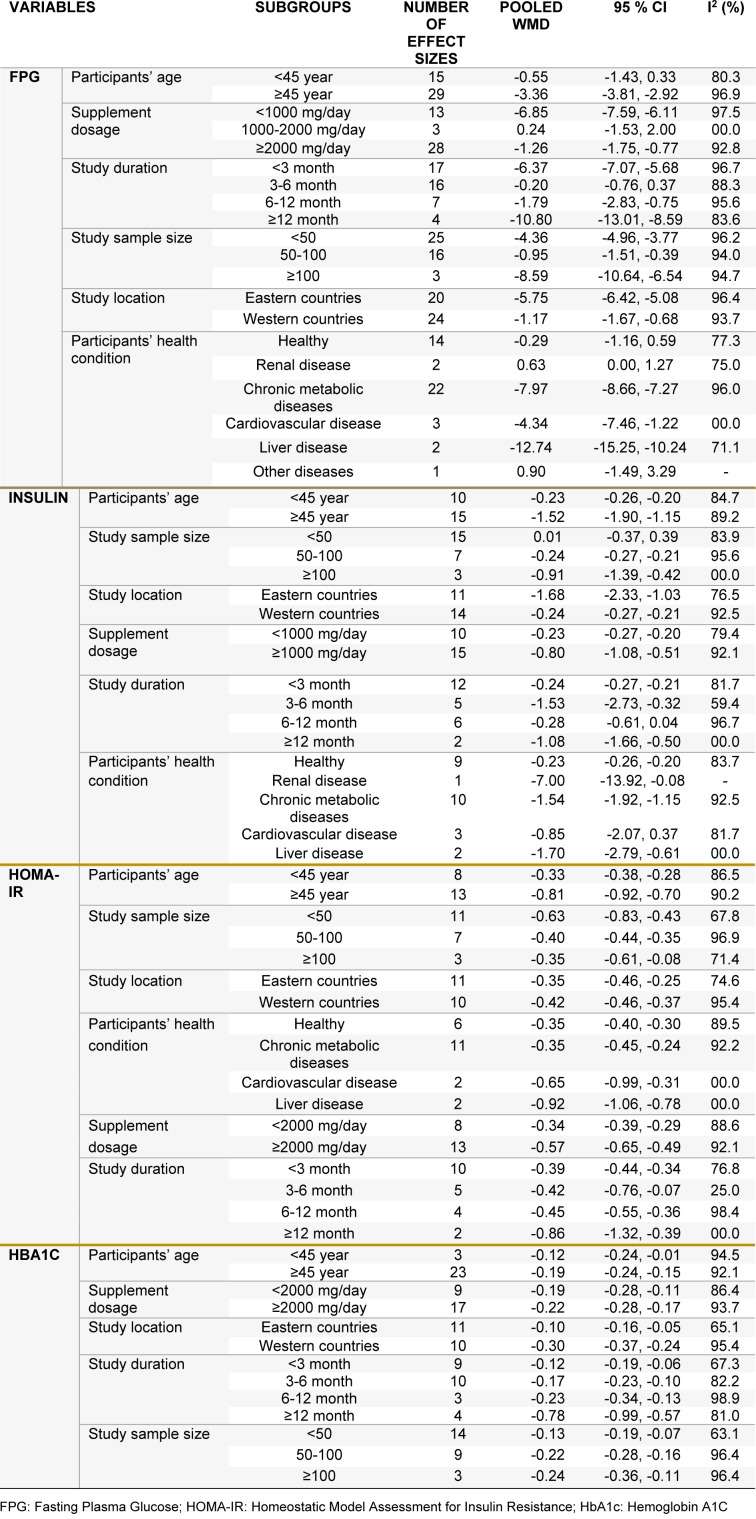
Subgroup analyses for the effects of carnitine supplementation on glycemic control
